# Fabrication of TiO_2_ Nanosheet Aarrays/Graphene/Cu_2_O Composite Structure for Enhanced Photocatalytic Activities

**DOI:** 10.1186/s11671-017-2088-7

**Published:** 2017-04-26

**Authors:** Jinzhao Huang, Ke Fu, Xiaolong Deng, Nannan Yao, Mingzhi Wei

**Affiliations:** 1 School of Physics and Technology, University of Jinan, Jinan, 250022 Shandong Province People’s Republic of China; 2 School of Material Science and Engineering, Qilu University of Technology, Jinan, 250353 Shandong Province People’s Republic of China

**Keywords:** TiO_2_ nanosheet arrays, Graphene, Cu_2_O, Heterostructure, Photocatalysis

## Abstract

TiO_2_ NSAs/graphene/Cu_2_O was fabricated on the carbon fiber to use as photocastalysts by coating Cu_2_O on the graphene (G) decorated TiO_2_ nanosheet arrays (NSAs). The research focus on constructing the composite structure and investigating the reason to enhance the photocatalytic ability. The morphological, structural, and photocatalytic properties of the as-synthesized products were characterized. The experimental results indicate that the better photocatalytic performance is ascribed to the following reasons. First, the TiO_2_ NSAs/graphene/Cu_2_O composite structure fabricated on the carbon cloth can form a 3D structure which can provide a higher specific surface area and enhance the light absorption. Second, the graphene as an electron sink can accept the photoelectrons from the photoexcited Cu_2_O which will reduce the recombination. Third, the TiO_2_ nanosheet can provide more favorable carrier transportation channel which can reduce the recombination of carriers. Finally, the Cu_2_O can extend the light absorption range.

## Background

Recently, application of semiconductor photocatalyst titanium dioxide (TiO_2_) in environmental purification has attracted great attention owing to its tremendous advantages, such as stability, nontoxicity, and low cost [1–3]. Based on the fact that photocatalytic reactions mainly take place on the surfaces of the photocatalysts, the morphology is the crucial factor to determine the efficiency [4]. Up to now, many efforts have been made to fabricate TiO_2_ nanocrystals, nanowires, nanorods, and nanotubes [5–8]. However, the application of TiO_2_ nanosheet arrays with larger specific surface area in photocatalytic degradation is rarely reported. Especially, TiO_2_ nanosheet arrays grown on the carbon cloth can construct three-dimensional (3D) structures to improve the specific surface area.

Unfortunately, the wide bandgap of TiO_2_ limits the effective absorption of visible light [9]. In order to overcome the shortcoming, one strategy is to modify TiO_2_ with narrow bandgap semiconductors [10–12]. Among them, cuprous oxide (Cu_2_O) with narrow bandgap can be a promising candidate for expanding the absorption spectra range. [13–18] Moreover, the built-in electric field between P-type Cu_2_O and N-type TiO_2_ can accelerate the separation of carriers.

However, the poor interfacial feature between different semiconductors directly influences the separation efficiency of carriers. Therefore, the interfacial optimization is an effective way to enhance photocatalytic degradation efficiency [19, 20]. The previous researches indicate that graphene (G) shows excellent interfacial optimization function between different semiconductors due to its high conductivity and two-dimensional structure, which facilitates the interfacial contact and carrier transportation [21–25]. However, it is difficult to make graphene well disperse between different semiconductors. In this paper, a modified method is applied to fabricate the homogeneously dispersed G between TiO_2_ nanosheet and Cu_2_O.

**Fig. 1:**
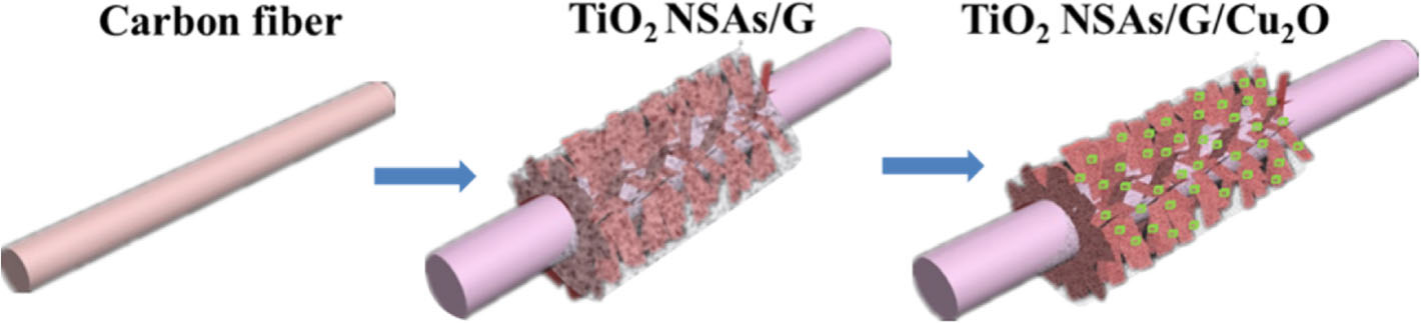
Schematic illustration for the preparation of TiO_2_ NSAs/G/Cu_2_O

In this study, the composite structure of TiO_2_ NSAs/G/Cu_2_O has been prepared (Fig. 1). The significant enhancement of photocatalytic activities was observed, and the corresponding results were analyzed. The proposed mechanism of the photocatalytic degradation was also discussed. As we know, there are no related reports on TiO_2_ NSAs/G/Cu_2_O photocatalysts up to now, so and thus it will be a meaningful reference for designing and fabricating this kind of photocatalyst used in photocatalytic degradation.

## Methods

### Preparation of TiO_2_ NSAs/G/Cu_2_O

The fabrication of TiO_2_ NSAs is the following. TiO_2_ sol was prepared using a previously reported method [10]. In brief, TiO_2_ seed layer was deposited on carbon cloth (2 cm × 3 cm) by immersing in TiO_2_ sol for 10 min. Then, the seed layer was calcined at 400 °C for 1 h. The Teflon-lined stainless steel autoclave (100 mL in volume) filled with 40 mL of aqueous solution of 10 M NaOH and 0.2 g of activated carbon was placed in an oven at 180 °C for 24 h. After the autoclave cooled down to room temperature, the prepare samples were rinsed with DI water to remove the residual activated carbon, followed by soaking with 0.1 M hydrochloric acid for 1 h, then washed to neutral with DI water.

For the composite structure of TiO_2_ NSAs/G, 0.2 g of graphene replacing activated carbon was added into the Teflon-lined stainless steel autoclave ethanol solution.

Cu_2_O layer was deposited by the following procedures. 2.3 mmol of Cu(CH_3_COO)_2_ and 2.3 mmol of CH_3_CONH_2_ were dissolved into 100 mL of diethylene glycol (DEG) under ultrasonication to prepare the reaction solution. Then, TiO_2_ NSAs or TiO_2_ NSAs/G substrate was immersed into the solution. Subsequently, it was heated to 120° under magnetic stirring and kept at this temperature for 6 h. After cooling down to room temperature in air, TiO_2_ or TiO_2_ NSAs/G substrate coated with Cu_2_O was washed with absolute ethanol and DI water for five times in sequence and dried in air.

### Characterization

The morphologies of the samples were investigated by a field emission scanning electron microscopy (FE-SEM, Quanta FEG250). The crystal structure of samples was examined by X-ray diffraction (XRD, D8 Advance) with Cu *K*_α_ at *λ* = 0.15406 nm radiation. XPS spectra were recorded on a Thermo Fisher ESCALAB 250Xi system with Al Kα radiation, operated at 150 W. The absorption spectrum of the samples was measured using a UV–vis spectrophotometer (TU-1901). The Raman spectrum of the sample was characterized by Raman spectroscopy (LabRAM HR800).

### Photoelectrochemical Measurement

Photocurrent density was measured using an electrochemical workstation (CS2350) in a three-electrode electrochemical cell with 1 M Na_2_SO_4_ as the electrolyte, in which the as-prepared samples were acted as the working electrode, Pt and Ag/AgCl electrode were used as the counter and reference electrodes, respectively. The *I*–*t* curve was recorded under Xe lamp (153 mW/cm^2^) irradiation.

### Measurement of Photocatalytic Activity

The photocatalytic activity was evaluated toward the photodegradation of RhB. A 500 W Xe lamp was used as the light source. The samples with same size 2 cm × 3 cm were placed into 20 ml RhB solution (10 mg/L). After irradiation for a designated time (30 min), 3 mL of RhB solution was taken out to identify the concentration of RhB using UV–vis spectrophotometer (TU-1901). All of these measurements were carried out at room temperature.

## Results and Discussion

**Fig. 2:**
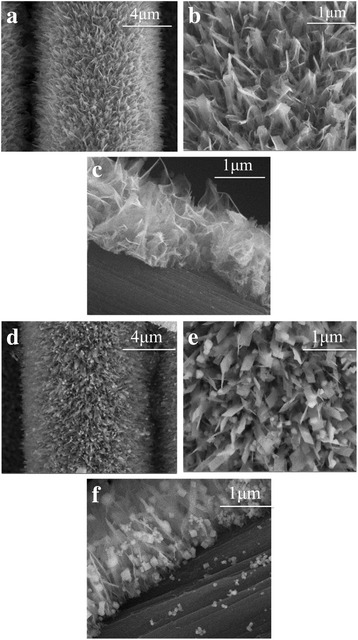
SEM images of **a**–**c** TiO_2_ NSAs with different magnification, **d**–**f** TiO_2_ NSAs/G/Cu_2_O with different magnifications

Figure 2a–c, the obtained TiO_2_ NSAs characterized by FE-SEM, shows that the uniform TiO_2_ NSAs with about 40–60 nm in width and 1.5 μm in height vertically grew on the surface of carbon cloth. These results illustrate that the morphology in favor of the enhancement of photocatalytic performance. As shown in Fig. 2d–f, Cu_2_O particles have been successfully deposited on the surface of the nanosheets. Unfortunately, G cannot be directly observed in Fig. 1d, e, though it could be confirmed by XPS measurement.

**Fig. 3:**
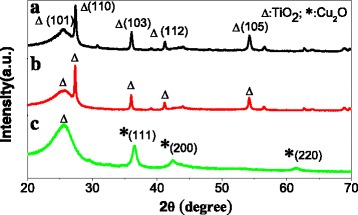
XRD patterns of **a** TiO_2_ NSAs, **b** TiO_2_ NSAs/G, and **c**TiO_2_ NSAs/G/Cu_2_O

XRD patterns were performed to investigate the crystal phase structure of photocatalyst. The XRD patterns of TiO_2_ NSAs, TiO_2_ NSAs/G, and TiO_2_ NSAs/G/Cu_2_O are shown in Fig. 3. For TiO_2_ NSAs (curve a), five distinctive peaks match well with TiO_2_. In curve b (for TiO_2_ NSAs/G), there are the same diffraction peaks with curve a. The phase on G is not detected because the content is low. From the curve c (TiO_2_ NSAs/G/Cu_2_O), the diffraction peaks on Cu_2_O are observed.

**Fig. 4:**
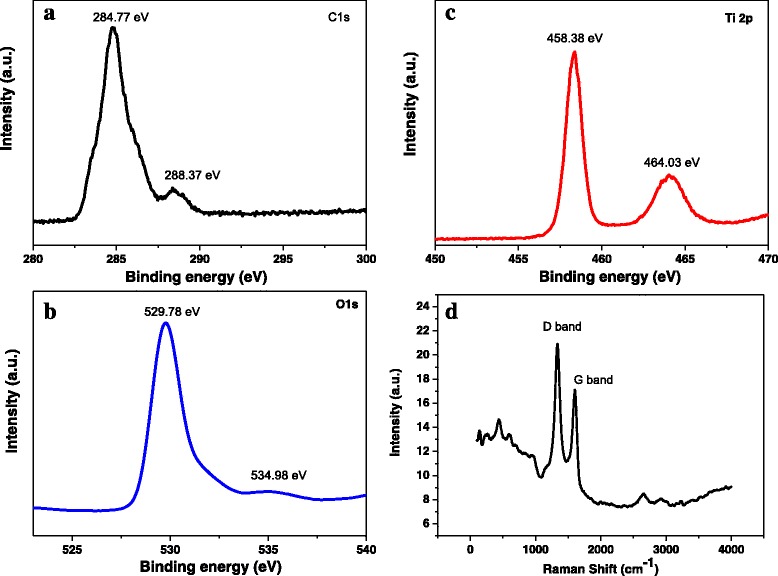
XPS spectra of **a** C 1s, **b** O 1s, **c** Ti 2p of TiO_2_ NSAs/G, and **d** Raman spectra of TiO_2_ NSAs/G

X-ray photoelectron spectroscopy (XPS) was used to confirm the existence of G. The XPS survey spectrum of TiO_2_ NSAs/G composite shows the elements C, O, and Ti (Fig. 4a–c). The presence of these elements proves the method to fabricate TiO_2_ NSAs/G composite is feasible. XPS spectrum of C1s located at 284.77 eV can be corresponded to carbon-containing species on the surface, which is the dominant existence form (Fig. 4a). Moreover, the 288.37 eV indicates the tiny existence of C–O bond. The peak located at 529.78 eV is related to the oxygen bonded with metal as Ti–O, and the 534.98 eV is the adsorbed oxygen or hydroxyl species (Fig. 4b). It can be seen that the spectra of catalysis showed two peaks at 458.38 and 464.03 eV. These peaks can be assigned to 3d5/2 and 3d3/2 spin orbital components of Ti^4+^ species (Fig. 4c) [26, 27]. In order to confirm the existence of graphene, furtherly, the Raman spectrum was characterized. The typical Raman spectra of TiO_2_ NSAs/G are shown in Fig. 4d. There are two typical Raman peaks corresponding to the typical D band and G band of graphene, respectively.

**Fig. 5:**
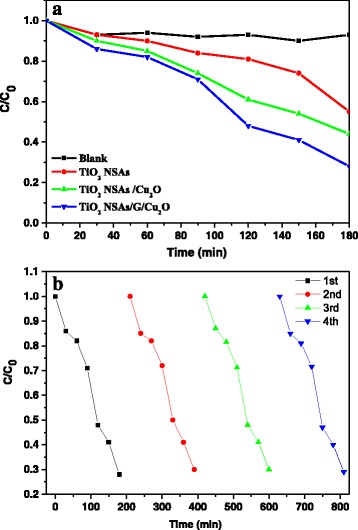
**a** Photocatalytic degradation of RhB in the presence of various catalysts. **b** Recycle of TiO_2_ NSAs/G/Cu_2_O under Xe lamp irradiation

The photocatalytic properties of the as-obtained samples were investigated by decomposition of RhB (Fig. 5a). Before irradiation started, the system was kept in dark for 1 h to reach the adsorption–desorption equilibrium. There is almost no change in the concentration of the solution when RhB solution is irradiated without any catalysts. After 180 min, the degradation ratio of RhB was almost 80% in the presence of TiO_2_ NSAs/G/Cu_2_O, whereas 50 and 40% of RhB was decomposed by TiO_2_ NSAs and TiO_2_ NSAs/ Cu_2_O, respectively. All of these measurements show that TiO_2_ NSAs/G/Cu_2_O exhibits more prominent photocatalytic activity compared with other samples. A very important reason for the advantage of TiO_2_ NSAs/Cu_2_O on the photodegradation of RhB is that Cu_2_O plays a significant role in extending light absorption spectrum. In addition, TiO_2_ NSAs/G/Cu_2_O exhibits a better photocatalytic activity than TiO_2_ NSAs/Cu_2_O which results from the presence of graphene. The graphene as an electron sink to accept the photoelectrons from the photoexcited Cu_2_O will reduce the recombination of photoelectron-hole pairs, resulting in a higher photocatalytic activity. The stability of the TiO_2_ NSAs/G/Cu_2_O was carried out, and the results (Fig. 5b) show that TiO_2_ NSAs/G/Cu_2_O has good stability.

**Fig. 6:**
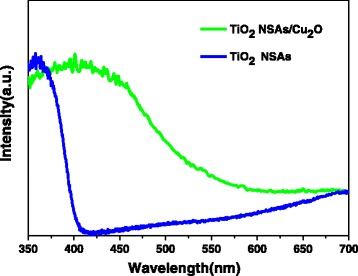
UV–vis absorption spectra of TiO_2_ NSAs and TiO_2_ NSAs/Cu_2_O

Generally, responding ability to light is one of the most important factors for evaluating photocatalytic performance. Therefore, UV–vis absorption spectra of samples were characterized as shown in Fig. 6. The absorption of TiO_2_ NSAs is located in the UV region. Compared with TiO_2_ NSAs, the absorption edge of TiO_2_ NSAs/Cu_2_O shows redshift. This larger absorption would result in the improvement of the photocatalytic property of TiO_2_ NSAs/Cu_2_O.

**Fig. 7:**
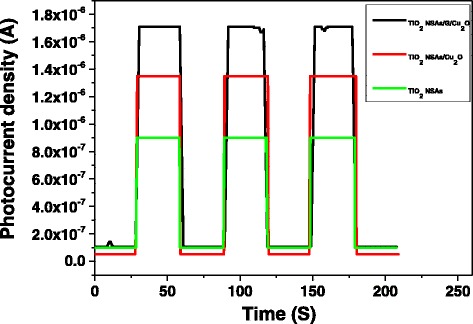
Photoinduced *I*–*t* curves of different samples

To further understand the improvement of photocatalytic activity, the *I*–*t* response of TiO_2_ NSAs, TiO_2_ NSAs /Cu_2_O, and TiO_2_ NSAs/G/Cu_2_O were observed, as shown in Fig. 7. It can be found that TiO_2_ NSAs/G/Cu_2_O exhibits enhanced photocurrents compared with TiO_2_ NSAs and TiO_2_ NSAs/Cu_2_O. The higher photocurrent density of TiO_2_ NSAs/G/Cu_2_O indicates an enhanced light absorption and higher separation efficiency of photogenerated electrons and holes.

The improved photocatalytic property of TiO_2_ NSAs/G/Cu_2_O may be attributed to the following factors. First, the introduction of Cu_2_O can extend the light absorption range, and thus, the photocatalytic activities are enhanced. Second, the limitation of carrier recombination is a key factor to enhance the photocatalytic property. The graphene as an electron sink can accept the photoelectrons from the photoexcited Cu_2_O which will reduce the recombination. Besides, TiO_2_ nanosheet structure can provide more favorable carrier transportation channel. Third, the better photocatalytic property can take advantage of large specific surface area. TiO_2_ NSAs/G/Cu_2_O structure fabricated on the carbon cloth can form a 3D structure which can provide a higher specific surface area. The high surface area of the 3D structure allows not only more surfaces to be reached by the incident light but also more sites on the surface for the adsorption and photodegradation of RhB, which results in enhanced photocatalytic performance. Finally, the 3D structure can enhance the photon utilization efficiency. The structure allows a great number of the photons to penetrate deep inside the photocatalyst, and most photons are trapped within the 3D structure until being completely absorbed.

**Fig. 8:**
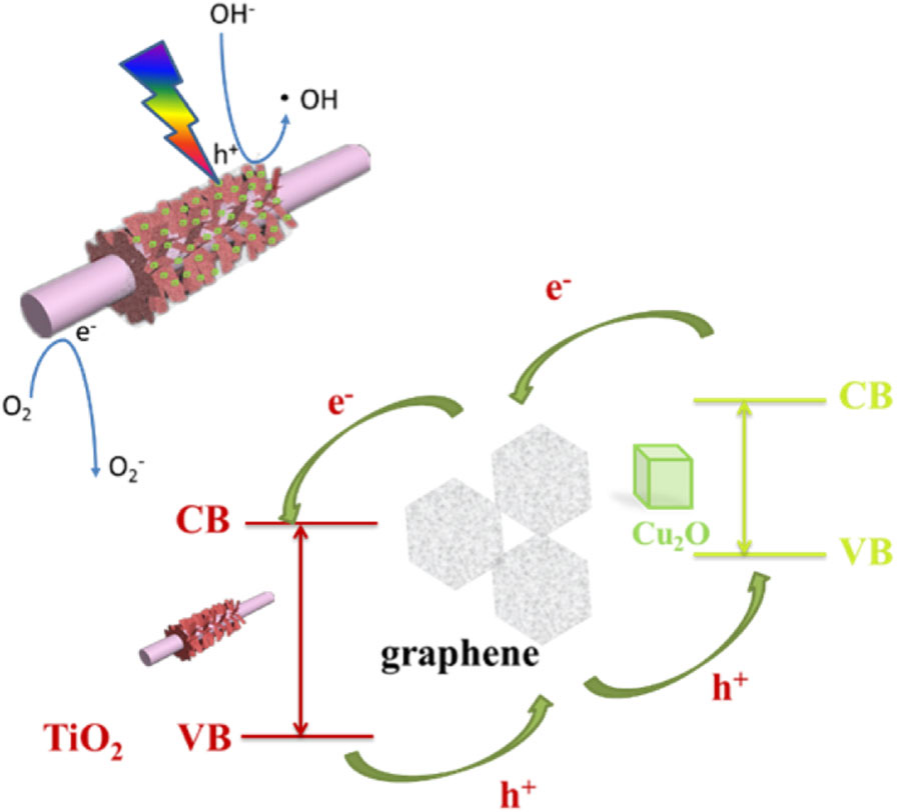
The possible photocatalytic mechanisms of TiO_2_ NSAs/G/Cu_2_O

The corresponding mechanism of electron transfer has been illustrated in Fig. 8. Both TiO_2_ and Cu_2_O can be photoexcited under the light irradiation. Because the *E*_VB_ of TiO_2_ is more positive than that of Cu_2_O, holes in the VB of TiO_2_ can migrate to the VB of Cu_2_O by the interface. Similarly, the *E*_CB_ of Cu_2_O is higher than that of TiO_2_, so the electrons in the CB of Cu_2_O can transfer to the CB of TiO_2_. More importantly, graphene as electronic exchange medium can promote electron transfer ability between Cu_2_O and TiO_2_.

## Conclusions

In summary, the novel 3D TiO_2_ NSAs/G/Cu_2_O structure is prepared via a simple and efficient method. Importantly, the composite structure exhibits excellent photocatalytic degradation properties. The enhanced performance can be ascribed to its extended light absorption range, large specific surface area, enhanced photon utilization efficiency, improved charge transfer efficiency and suppressed photoelectron-hole recombination. Furthermore, the photocatalysts grown on carbon cloths make the collection and recycle of photocatalysts much easier.
